# Video-based education improves the image quality of diagnostic percutaneous cerebral angiography among elderly patients

**DOI:** 10.1515/tnsci-2020-0107

**Published:** 2020-09-28

**Authors:** Wenbing Wang, Yongshun Wu, Jianpeng Yuan, Qian Yang, Zhiming Zhou

**Affiliations:** Department of Neurology, The First Affiliated Hospital, Yijishan Hospital of Wannan Medical College, Wuhu 241000, China; Department of Radiology, The Seventh Affiliated Hospital of Sun Yat-sen University, Shenzhen 518000, China

**Keywords:** video, education, DSA, image quality, elderly

## Abstract

**Objective:**

Digital subtraction angiography (DSA) is considered the gold standard for cerebral vasculature observation and is increasingly applied among the elderly population. The aim of this study is to determine whether the use of a video-based education system can improve the image quality of percutaneous cerebral angiography.

**Method:**

This study is a single-blinded prospective cohort trial. One hundred and sixty patients (≥65 years old) were enrolled in this study. Eighty patients were provided with video-based education as intervention. Eighty age-matched controls only received regular education. The DSA image quality was assessed between control and intervention groups. It was rated by two readers on a 5-point scale, independently.

**Results:**

No differences were found between control and intervention groups in baseline characteristics (*P* > 0.05). The mean overall image quality was significantly higher in patients receiving video-based education than in controls (*P* < 0.05), and the same trends were found in the respective assessment of each artery (left and right carotid/vertebral artery; *P* < 0.05). Moreover, the operation time and radiation doses were quite comparable between the two groups (*P* > 0.05).

**Conclusions:**

This study indicated that video-based education helps elderly patients to acquire improved DSA image quality. It encourages the application of this approach in practice.

## 1 Introduction

Elderly population is more likely to develop cerebral atherosclerosis diseases [1]. Digital subtraction angiography (DSA) is considered the gold standard for cerebral vasculature observation [2]. It is the first step during neurointerventional procedures, for example, endovascular thrombectomy and carotid artery stenting [3,4]. However, patients sometimes cannot cooperate with the operation process, keeping still when they receive radiological assessment, for example, magnetic resonance imaging (MRI) [5,6]. This may be due to the unfamiliar environment, the fear of potential risks, and unawareness of procedure details [7,8]. All these would make patients undergo excess motions. We then assumed that the excess motion also occurs in DSA and subsequently influences the acquirement of high-quality images.

It is believed that preprocedure training helps relieve patients from the anxiety condition and improve the results of diagnostic and therapeutic approaches [9,10,8]. Patients are usually provided with training to know procedure details by literal, verbal and graphic manners [8]. Researchers have indicated that video is more comprehensive and acceptable [11,12] for examinees to understand the theory and operation details of a certain approach. Moreover, emerging attention is paid to radiation generated in DSA. The unwanted radiation exposure during DSA may increase the risk of skin damage among examiners and examinees, especially in the case of a prolonged examination process [13,14]. Strategies concerning the reduction of DSA radiation are constructed in recent years.

A video-based training education approach is introduced in this study to evaluate its effects on image quality and operational outcomes in diagnostic cerebral catheter angiography.

## 2 Materials and methods

### 2.1 Study population

**Figure 1:**
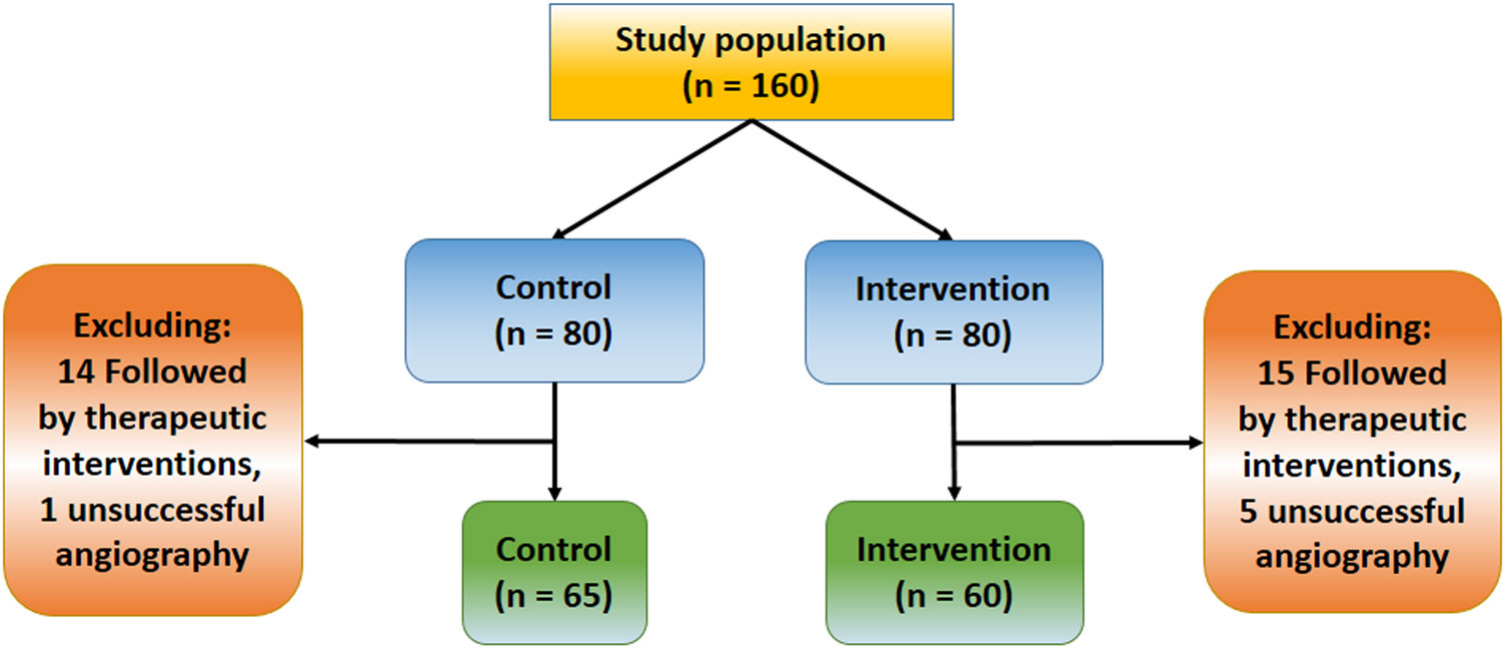
Participant flow chart of the study.


This study was conducted in Neurointerventional Center of Yijishan Hospital, Wannan Medical College from March 1, 2018, to September 1, 2018. This center is a tertiary 3,000-bed hospital, serving the population of about 30,00,000 in the southern part of Anhui Province. Eighty patients were provided with video-based education, and 80 age-matched patients with general education were enrolled in this study. Patients should fulfill the following requirements: (1) older than 65 years; (2) receiving the examination for the first time, without knowledge of DSA details before enrollment; (3) speaking Mandarin Chinese or local dialect and (4) owning an educational background of high school at least. Patients were excluded if they had cognitive impairment, were unwilling to participate in the study or lack communication skills. Data were removed if a patient finally had to receive therapeutic interventions for stenosis and aneurysm changes in arteries according to the results of diagnostic DSA. A flow diagram of the inclusion process of the study is shown in Figure 1.

**Ethical approval:** The research related to human use has been complied with all the relevant national regulations, institutional policies and in accordance with the tenets of the Helsinki Declaration and has been approved by the Ethics Committee of Wannan Medical College.
**Informed consent:** Informed consent has been obtained from all individuals included in this study.

### 2.2 Education system

One day before examination, patients in the intervention and the age-matched control would receive a brief handbook introducing examination machines and procedural details of diagnostic and therapeutic DSA in the form of texts and pictures. Then, an experienced physician blind to the study explained the details and answer questions from the patients. The physician would also tell the subjects the possibility and the processes of further therapeutic interventions based on the angiographic results. Besides, patients in the intervention arm would receive a video-based education. This video vividly describes the purpose, the whole processes of DSA procedure and a brief introduction of therapeutic interventions, with a length of 3 min. Subsequently, the same physician was available for consultation. Finally, patients in both control and intervention groups were guided to the operation room to gain familiarity with the entire procedure and the medical environment for 10–15 min. Familiarization involved showing the parts of the machines and the operation room. In addition, patients were told about the importance of minimizing motion while the scan was conducted. Several images were shown highlighting the effects of motion on image quality.

### 2.3 Diagnostic DSA procedure

Diagnostic cerebral DSA was performed using a biplane flat detector angiography suit (Artis Zee Flat Detector Biplane Angiosuite, Siemens). The system supported Digital Imaging and Communications in Medicine Radiation Dose Structured Reports. Standard dose imaging was done using the protocol defined by the manufacturer with the detector dose of 3.6 µGy/frame. All procedures were performed with systemic anticoagulation using heparin with an initial 3,000 U bolus, followed by an intraoperational re-bolus of 1,000 U at each additional hour. Percutaneous access was gained through the femoral artery employing the Seldinger technique. 6 F short sheaths were inserted into the artery. A guiding catheter (5 F) was advanced into the common carotid artery through a long 0.035-inch support wire. The aortic arch was imaged in the left anterior-oblique and anteroposterior view with 35–40 mL of contrast medium at an infusion rate of 15 mL/s. Six to nine milliliters of contrast medium was used per acquisition, consisting of one anteroposterior, one lateral and two oblique views of both internal carotid artery (ICA)s and vertebral artery (VA)s at the injection rate of 4–6 mL/s. DSA shots were acquired at 4 frames per second to a late venous phase by using a 36-cm^2^ field of view, pixel size 0.15 × 0.15. Magnification views were done if necessary to clarify suspected changes. The intracranial circulation was included in all acquisitions. Rotational angiography was used when needed. After the procedure, patients were transferred to the neurology ward with continuous ECH monitoring for the following 24 h. Noninvasive blood pressure measurements were done every 0.5 h for the initial 4 h and every 2 h for the following 20 h.

### 2.4 Image quality

**Figure 2:**
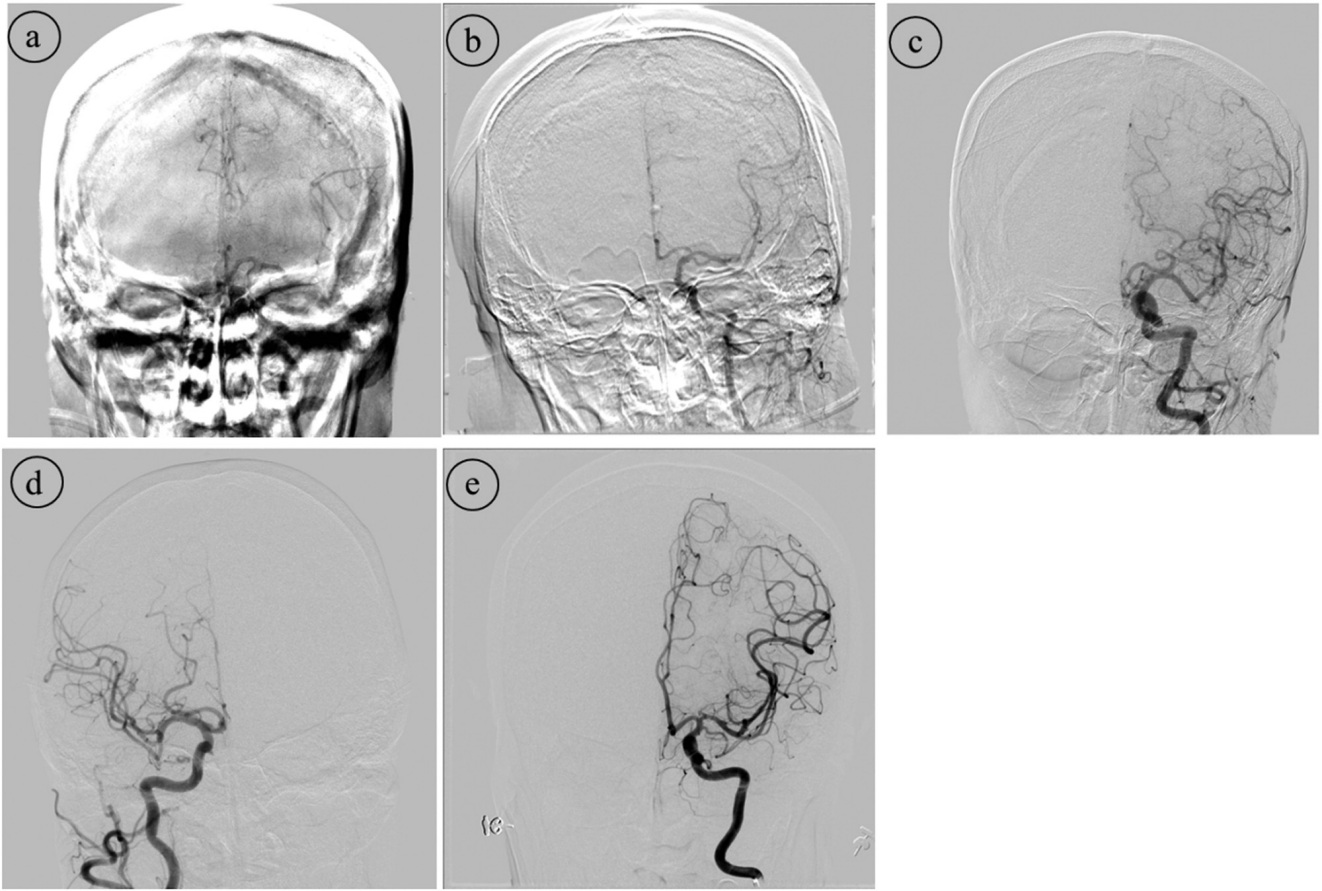
DSA image quality score with picture illustration.

Images of ICAs and VAs in each individual were obtained for the quality assessment of anteroposterior and lateral DSA images, respectively. These images were excluded from assessment if any flow-limiting vascular stenosis/stenosis or artery steal phenomenon from arteriovenous shunts obscured relative opacification of a vessel segment or distribution. All analyses were performed in consensus by two independent neuroradiologists, each of whom has more than 5 years of clinical experience in cerebral imaging, on a system workstation (Siemens), without knowledge of the study design. Each DSA image was defined with respect to arterial, capillary and venous phases, determined by a 5-point scale on DSA images [15] (Table 1 and Figure 2) and followed by an average value of the total cumulative score.

**Table 1:** The quality scale of DSA images

Quality	Definition
5	Very good, excellent large and small vessel visualization
4	Good, excellent large vessel and minimal compromise of small vessel visualization
3	Average, diagnostic value for large vessel, but compromised small vessel visualization
2	Poor, compromised large and small vessel visualization
1	Bad, cannot be used for diagnosis

DSA, digital subtraction angiography.

### 2.5 Data collection

Data on patient demographics, procedural equipment and technical details were collected. Radiation and time parameters were collected after every DSA event from machine and recorders’ reports, including radiation time, kerma air product (KAP), number of images, number of DSA shots and each shot duration.

### 2.6 Statistical analysis

Data were computerized and analyzed with the use of SPSS 21.0 software package. Data were summarized using frequency tables for categorical variables. Mean ± standard deviation (SD) was calculated for continuous parameters. All *P* values were two sided, and *P* < 0.05 was set as statistical significance.

## 3 Results

### 3.1 Baseline characteristics

A total of 160 patients participated in this study and were allocated into control (*n* = 80) and intervention groups (*n* = 80) (Table 2). Thirty-three patients were excluded finally, of whom 29 received therapeutic approaches based on angiographic results (14 in control and 15 in intervention), and four proved to have unsuccessful angiography. Finally, there were 65 in control group (aged 70.65 ± 4.72 years) and 60 in intervention group (68.57 ± 7.80 years). There were no significant differences between the two groups with respect to age, gender, disease history and arch type (*P* > 0.05).

**Table 2:** Baseline characteristics of the participants

Variables	Control (*n* = 65)	Intervention (*n* = 60)	*P* value
Age	70.65 ± 4.73	68.57 ± 7.80	0.078
Male	41	38	0.562
Disease history			
Stroke	31	33	0.475
Smoking	14	19	0.227
Alcohol	7	10	0.436
Diabetes	15	19	0.318
Hypertension	46	40	0.700
Hyperglycemia	7	9	0.595
Arch type			0.311
I	27	20	
II	27	33	
III	11	7	

### 3.2 Image quality analysis

As presented in Tables 3, three patients in intervention group and one patient in control group were excluded in the image quality analysis, failing to export data from the working station. A careful review of the rest records revealed that video education before angiography induced a higher overall score of image quality than control, with respect to all arteries and overall image scores (*P* < 0.05).

**Table 3:** Comparison of image quality between control and intervention

	Image quality				
Group (control/intervention)	Left ICA	Right ICA	Left VA	Right VA	Overall (64/57)
Control	3.65 ± 0.66	3.54 ± 0.58	3.26 ± 0.69	2.84 ± 0.78	3.33 ± 0.52
Intervention	4.06 ± 0.54	3.83 ± 0.59	3.60 ± 0.75	3.34 ± 0.70	3.72 ± 0.49
*P* value	<0.0001	0.006	0.012	0.001	<0.0001

ICA, internal carotid artery; VA, vertebral artery.

The kappa consistency test values of the scores between the two observers were as follows: left internal carotid artery, 64.4%; right internal carotid artery, 62.8%; left vertebral artery, 67.9%; right vertebral artery, 68.3%.

### 3.3 Operation analysis

We found comparable results in time parameters between the two groups: operation time, DSA shot time and fluoroscopy time (*P* > 0.05; Table 4). Also, there were no significant differences in KAP, DSA shot dose, fluoroscopy dose and cumulative dose (*P* > 0.05).

**Table 4:** Comparison of radiation and time parameters between control and intervention

Variables	Control	Intervention	*P* value
Time			
Operation time	53.44 ± 22.68	52.74 ± 17.29	0.848
DSA shot	94.48 ± 32.10	94.02 ± 29.42	0.933
Fluoroscopy	656.51 ± 591.16	580.69 ± 333.91	0.375
KAP	18611.69 ± 7184.22	19176.75 ± 5612.53	0.706
Radiation dose			
DSA shot	652.04 ± 214.95	684.22 ± 194.33	0.381
Fluoroscopy	131.91 ± 169.32	107.92 ± 71.63	0.299
Cumulative	783.95 ± 299.34	792.14 ± 241.07	0.866

DSA, digital subtraction angiography; KAP, kerma area product.

## 4 Discussion

The main finding of our study demonstrated the substantially improved image quality of diagnostic percutaneous cerebral angiography among elderly participants by an innovative video-based education system. However, no obvious changes in radiation and time parameters generated in the operation were observed by education intervention.

The index results are in line with previous studies, which have indicated that the education approach and familiarization with medical equipment and technics can greatly improve the performances of diagnostic and therapeutic approaches [16,17,18,19]. For example, a recent study by Ahlander *et al.* [20] indicated that video given before cardiovascular MRI helps patients relax during the imaging. Hayat *et al.* [17] found that a patient-centered educational video improves bowel preparation quality and may reduce the possibility of a repeat procedure in patients with screening colonoscopy. Also, the usefulness of a preparation program among children before MRI reduces the sedation use, increases the successful scanning and obtains more acceptable images for diagnosis [21,22]. However, no studies were available for the analysis of the education system on performances of DSA, especially on elderly ones. Elderly people are more likely to suffer from cerebrovascular diseases than younger ones, such as ischemic stroke and severe and accelerated atherosclerosis [23,24]. When faced with the comprehensive introduction of DSA technology, operation details and importance of keeping still, participants, who viewed an educational video in this study, were more likely to perform better cooperation during the examination to acquire a better quality of images. This work extended and build on these studies by demonstrating the efficacy of the video support tool to assess the DSA image quality using a 5-point scale with respect to the overall visualization of large and small vessels [15]. Most importantly, this approach succeeded in acquiring a better quality of DSA images with its help.

Percutaneous cerebral angiography helps understand the pathological changes of the cerebral circulation and emerges as a new therapeutic method for cerebral stenosis and aneurysms [25,26]. It is being applied in more people. But it is essential to acquire high-quality images that the examinees should keep still during the procedure. As a newly spreading method, it is unfamiliar to many participants. Sometimes, it may be hard for them to understand the necessity of keeping still. Also, the pains, anxiety and fears during the examination may induce excess motility, causing a possible impairment of image quality and prolonged examination. It is believed that video is more capable of sending information and improving the receivers’ understanding of messages [11]. The introduction of video in the intervention group in the study emphasized the importance of it and strongly recommended it to the subjects. It might help reduce anxiety and fears among patients, thus assisting them in keeping still. This may be the cause of high-quality images acquired in subjects receiving education.

Moreover, researchers have consistently shown their concerns on the radiation outcomes during DSA. The lengthy procedure may increase the radiation on both examiners and examinees, causing an increased risk of skin diseases [13,14]. Our center is also focusing on radiation risk of subjects receiving percutaneous angiography [2]. We have confirmed the efficacy of the video-based education approach in radiation and operation parameters. However, no reduction of radiation parameters and time parameters in all aspects were induced.

Our study has several limitations. First, this is a relatively small study recruiting patients who were all Chinese, well educated and drawn from one teaching hospital. We then cannot say that the findings could also be generalizable to those less educated ones. Education level or cultural background may influence the understanding and cooperation among subjects. Second, the video described both diagnostic DSA and further therapeutic interventions, along with its operation risks. This may increase the anxiety among the participants in a relatively “vivid” way. We were unable to assess whether and how this approach would affect the emotional responses of patients. Third, we analyzed intracranial cerebral blood flow images in the study. The effects of education on image quality of vasculature in other body parts were not studied.

## 5 Conclusion

The video-based education approach helps improve the image quality and reduces the operation time as well as radiation dose in diagnostic DSA. It should be encouraged in the elderly population in clinical practice.
